# Regiospecific C–H amination of (−)-limonene into (−)-perillamine by multi-enzymatic cascade reactions

**DOI:** 10.1186/s40643-022-00571-x

**Published:** 2022-08-26

**Authors:** Yue Ge, Zheng-Yu Huang, Jiang Pan, Chun-Xiu Li, Gao-Wei Zheng, Jian-He Xu

**Affiliations:** Laboratory of Biocatalysis and Synthetic Biotechnology, State Key Laboratory of Bioreactor Engineering, Shanghai Collaborative Innovation Centre for Biomanufacturing, College of Biotechnology, East China University of Science and Technology, Shanghai, 200237 People’s Republic of China

**Keywords:** CYP153A7, (−)-Limonene, Multi-enzyme cascade, Regiospecific C–H amination, (−)-Perillamine, Transaminase

## Abstract

**Background:**

(−)-Limonene, one of cyclic monoterpenes, is an important renewable compound used widely as a key building block for the synthesis of new biologically active molecules and fine chemicals. (−)-Perillamine, as derived from (−)-limonene, is a highly useful synthon for constructing more complicated and functionally relevant chemicals.

**Aim:**

We aimed to report a more sustainable and more efficient method for the regiospecific C–H amination of (−)-limonene into (−)-perillamine.

**Results:**

Here, we report an artificial penta-enzymatic cascade system for the transformation of the cheap and easily available (−)-limonene into (−)-perillamine for the first time. This system is composed of cytochrome P450 monooxygenase, alcohol dehydrogenase and w-transaminase for the main reactions, as well as formate dehydrogenase and NADH oxidase for cofactor recycling. After optimization of the multi-enzymatic cascade system, 10 mM (−)-limonene was smoothly converted into 5.4 mM (−)-perillamine in a one-pot two-step biotransformation, indicating the feasibility of multi-enzymatic C7-regiospecific amination of the inert C–H bond of (−)-limonene. This method represents a concise and efficient route for the biocatalytic synthesis of derivatives from similar natural products.

**Supplementary Information:**

The online version contains supplementary material available at 10.1186/s40643-022-00571-x.

## Introduction

Monoterpenoids exist widely in the secretory tissues of plants, most of which have strong aroma and physiological activity, such as anti-inflammatory, anticonvulsant, and antiviral activities, and are widely used as starting scaffolds in the synthesis of biologically active compounds (Fordjour et al. 2022; Huang et al. 2021; Salakhutdinov et al. 2017). Limonene, naturally present in herbal essential oil, has pathogen-selective antimicrobial activity (da Silva et al. 2021; Du et al. 2014). The enantiomeric configuration influences the biological activity of limonene, and (−)-limonene is more active than (+)-limonene (van Vuuren and viljoen 2007). (−)-Perillyl alcohol, a derivative of (−)-limonene, has great efficacy in the treatment of a variety of tumors (Hui et al. 2014). However, the results in clinical trials are not ideal. Thus, further structural optimization has become the direction of new drug development researches. Several studies on monoterpenoids have shown that amino-modification can improve their solubility and anti-tumor activity (Chen et al. 2006; Xu et al. 2006).

The creation of hybrid molecules from natural compounds is a modern and a highly relevant trend in medicinal chemistry (Luzina et al. 2020). (−)-Perillamine plays an important role as an intermediate and building block molecule. In recent years, Suslov et al. synthesized a series of amides by combining the adamantane and monoterpene moieties to search for novel low molecular weight orthopoxvirus inhibitors (Suslov et al. 2020). A series of combined fragments of usnic acid and monoterpenoids were synthesized with (−)-perillamine by Dyrkheeva et al., affording effective tyrosyl-DNA phosphodiesterase I inhibitor for anticancer therapy in combination with clinically anticancer drug topotecan (Dyrkheeva et al. 2021). These studies highlight the tremendous application value of (−)-perillamine, indicating that the efficient synthesis of (−)-perillamine is of great significance to the development of new biologically active compounds.

The chemical synthesis of (−)-perillamine needs expensive catalysts and harsh reaction conditions (Dyrkheeva et al. 2021; Suslov et al. 2020). Therefore, it is of great importance to develop a more sustainable and more efficient method for the regioselective amination of inert C–H bonds. Biocatalysis has been developed as an efficient tool to enrich, expand or even replace the traditional chemical synthesis methods for pharmaceutical industry due to its excellent selectivity, mild reaction conditions and environmental friendliness. The biological cascade reaction combines two or more catalysts to catalyze the completion of a series of continuous chemical reactions (Bruggink et al. 2003). On the one hand, the cascade reaction has higher atomic economy and can save reaction space and time; on the other hand, the cascade reaction does not need to isolate and purify the intermediate products after each reaction step, which can effectively improve the utilization rate of the substrate and the yield of the terminal product (Jiang et al. 2021). In this study, a multi-enzyme cascade reaction system was designed and built to realize regiospecific C–H amination of (−)-limonene at C-7 position for facile and efficient synthesis of the biologically active compound (−)-perillamine, by use of P450 hydroxylase, alcohol dehydrogenase, and transaminase.

## Methods

### Chemicals and materials

(−)-Limonene, (−)-perillyl alcohol and (−)-perillyl aldehyde were purchased from Energy chemical Ltd. (Shanghai, China). All other chemicals of analytical grade or higher purity were obtained from commercial sources without further purification. Tryptone and yeast extract were obtained from Oxoid (Hampshire, UK). Genes were synthesized by Genscript Biotech Co. Ltd. (Nanjing, China). Formate dehydrogenase from *Candida boidinii* (*Cb*FDH), alcohol dehydrogenase from *Lactobacillus kefiri* (*Lk*ADH), NADH oxidase from *Streptococcus mutans* (*Sm*NOX) and w-transaminase from *Arthrobacter* sp. (ATA-117) were obtained from the enzyme library deposited in our laboratory.

### Gene cloning and expression

The genes encoding *Lk*ADH, *Sm*NOX and ATA-117 were, respectively, ligated into pET-28a and pET-21a under the control of T7 promoter and then transformed into *E. coli* BL21 (DE3) for overexpression. Positive transformants and glycerin strains were grown at 37 °C to an optical density at 600 nm of 0.6–0.8 in Terrific broth liquid medium containing 50 μg/mL ampicillin for pET-21a (or kanamycin for pET-28a). Protein production was induced by adding isopropyl-b-d-thiogalactopyranoside (IPTG) to a final concentration of 0.2 mM, and the cells were cultured for a further 22 h at 16 °C and 220 rpm. The protein production of P450pyr required extra supplement of 5-aminolevulinic acid (ALA) to a final concentration of 0.5 mM.

### Lyophilized enzyme powders preparation of *Lk*ADH, *Sm*NOX and ATA-117

The *Lk*ADH, *Sm*NOX and ATA-117 enzymes were overexpressed as described above. Cells were harvested by centrifugation (10,000×*g*) at 4 °C for 10 min, resuspended in KPB buffer (10 mM, pH 7.5). A clear lysate was obtained by high-pressure homogenizer and subsequently centrifuged (10,000×*g*) for 40 min to remove cell debris. The supernatant was frozen at − 80 °C overnight and then lyophilized to obtain the cell-free enzyme powders.

### Enzyme assay

The specific activities of *Lk*ADH and *Sm*NOX were measured by monitoring the initial change of the absorbance of nicotinamide cofactors at 340 nm (*e* = 6220 M^−1^ cm^−1^) and 30 °C using a UV–Vis spectrophotometer (Shimadzu, Japan). For *Lk*ADH, reactions were performed in a 1 mL cuvette supplemented with KPB buffer (100 mM, pH 7.5) containing 10 mM (−)-perillyl alcohol, 1 mM NAD^+^, and an appropriate amount of enzyme. For *Sm*NOX, reactions with oxygen-saturated KPB buffer (100 mM, pH 7.5) containing 0.2 mM NADH.

The transamination activity of w-TA was measured by gas chromatography (GC) with (−)-perillyl aldehyde as substrate. Reactions were performed in 2-mL Eppendorf tube with 500-µL reaction system containing 10 mM (−)-perillyl aldehyde, 80 mM 2-pentanamine, 0.2 mM pyridoxal-5-phosphate (PLP) and an appropriate amount of enzyme for 45 min at 35 °C. Determination of product concentration was done using GC to calculate the specific activities of ω-TA.

One unit of the enzymatic activity was defined as the amount of enzyme catalyzing the reduction (or oxidation) of 1 μmol of the substrate in 1 min.

### Analytical methods

The reaction solution (500 μL) was extracted with 500 μL methyl *tert*-butyl ether (MTBE) containing camphor as an internal standard and dried over anhydrous Na_2_SO_4_ for 6 h for GC analysis. (−)-Limonene, (−)-perillyl alcohol, (−)-perillyl aldehyde and (−)-perillamine were measured by Shimadzu-2014 gas chromatography equipped with a DB 1701 column (30 m × 0.25 mm, 0.25 μm). The temperature for the injector and detector was 250 °C, and the column temperature was first set at 80℃ for 2 min, raised to 200 ℃ at 5 ℃ min^−1^ and held for 2 min. The inlet volume of the sample was 1 μL and the split ratio was 20:1.

### Biocatalytic cascade reaction in one pot

The analytical-scale one-pot reaction mixture (0.5 mL) contained 10 mM (−)-limonene (with 2% v/v DMSO), 15 g_cdw_/L A7F resting cells, 100 mM sodium formate, 1 mg/mL Triton X-100, and KPB buffer (100 mM, pH 7.5). The reaction mixtures were shaken at 25 °C and 800 rpm for 2 h. After the reaction mixture was heated at 65 °C in a water bath for 0.5 h and cooled to room temperature, the Module-2 reaction was initiated by supplementing 0.2 U mL^−1^ *Lk*ADH, 1.0 U mL^−1^ *Sm*NOX, 2 U mL^−1^ ATA-117, 0.2 mM NAD^+^, 0.2 mM PLP, and 80 mM 2-pentanamine and continued by incubation at 35 °C for 12 h. To terminate the reaction, was added 10 μL NaOH solution (10 M) before the reaction mixture was extracted by 0.5 mL MTBE. The organic extract was dried with anhydrous Na_2_SO_4_ and analyzed by GC.

### Preparative-scale reaction

A preparative-scale mixture (100 mL), containing 100 mM KPB buffer (pH 7.5), 10 mM (−)-limonene, 15 g_cdw_ L^−1^ A7F resting cells, 0.2 mM NAD^+^, 100 mM sodium formate, 1 mg/mL Triton X-100, was shaken at 25 °C and 200 rpm for 2 h. After the hydroxylation step was completed, the reaction mixture was heated at 65 °C in a water bath for 35 min, and then cooled to room temperature. Subsequently, all the elements desired for Module 2, including 0.2 U mL^−1^ *Lk*ADH, 1.0 U mL^−1^ *Sm*NOX, 2 U mL^−1^ ATA-117, 0.2 mM NAD^+^, 0.2 mM PLP and 80 mM 2-pentanamine, were supplemented into the reaction system and agitated magnetically at 35 °C for 12 h. Then 2 mL NaOH solution (10 M) was added to terminate the reaction before being extracted with MTBE. The organic extract was dried over anhydrous Na_2_SO_4_. The final precipitate was evaporated again under reduced pressure and dried under vacuum to give (−)-perillamine.

## Results and discussion

### Design of a modularized multi-enzymatic cascade for converting (−)-limonene into (−)-perillamine

**Scheme 1:**
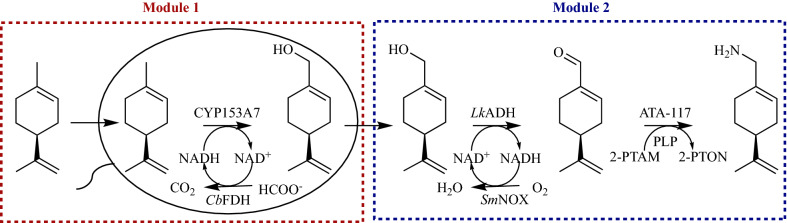
A multi-enzyme cascade designed for the regiospecific bioamination of (−)-limonene into (−)-perillamine. *2-PTAM* 2-pentanamine, *2-PTON* 2-pentanone

In the cascade process, (−)-limonene was initially hydroxylated by a cytochrome P450 enzyme to produce (−)-perillyl alcohol. The (−)-perillyl alcohol was further oxidized by an alcohol dehydrogenase (ADH) to produce (−)-perillyl aldehyde and then aminated by an w-transaminase (w-TA) to give (−)-perillamine simultaneously. We tried to recycle the effective nicotine cofactor by introducing a formate dehydrogenase (FDH) and an NADH oxidase (NOX) for regenerating the desired NADH (for C–H monooxygenation) and NAD^+^ (for alcohol dehydrogenation), respectively. Considering the instability of P450 enzymes and the interference between the two coenzyme recycling systems which might result in a thermodynamic balance, we divided this cascade into two independent modules (Module 1 and Module 2). Module 1 was composed of a P450 enzyme and an FDH for effectively hydroxylating (−)-limonene to (−)-perillyl alcohol, while Module 2 comprises an ADH, an NOX and an w-TA to transform the (−)-perillyl alcohol into (−)-perillamine (Scheme 1).

### Recruiting enzymes for constructing Module 1

Terpenoid hydroxylases are mainly cytochrome P450 enzymes, which have significant substrate promiscuity. According to the type of reaction catalyzed by the enzyme and the structure of substrate, we selected several terpene hydroxylases for the first hydroxylation step. Through functional characterization, we found that CYP153A7 could catalyze the regiospecific hydroxylation of (−)-limonene into (−)-perillyl alcohol. CYP153A7, originated from *Sphingomonas* sp. HXN-200, is a class I P450 enzyme with a broad substrate scope and applications (Chang et al. 2000, 2002a, b; Dong et al. 2022; Li et al. 2001). It requires ferredoxin (Fdx) and ferredoxin reductase (FdR) for electron transfer and can be used for the regio- and stereo-selective hydroxylation of non-activated carbon atoms (Pham et al. 2012; Yang and Li 2015). Therefore, we utilized an engineered *E. coli* BL21(DE3) strain (A7F), constructed previously in our laboratory, which co-expresses the CYP153A7 gene with its redox partners (Fdx & FdR) and a formate dehydrogenase from *Candida boidinii* (*Cb*FDH) (Hummel 1999; Slusarczyk et al. 2000; Tang et al. 2021) for the effective cofactor regeneration.

### Recruiting enzymes for constructing Module 2

Considering the high similarity of the chemical structures between (−)-perillyl alcohol and *p*-mentha-1,8-dien-3-ol, we tested the toolbox of *p*-mentha-1,8-dien-3-ol dehydrogenases preserved in our laboratory and found that a double mutant of alcohol dehydrogenase, *Lk*ADH_T91F/I195V_ from *Lactobacillus kefiri* (*Lk*ADH), could catalyze the oxidation of (−)-perillyl alcohol into (−)-perillyl aldehyde. In order to regenerate the consumed cofactor NADH, we introduced a variant of the highly efficient NADH oxidase, *Sm*NOX_V193R/V194H_ from *Streptococcus mutans* (*Sm*NOX) (Jiao et al. 2016). Since many w-TAs were reported for the transamination of aldehydes, we first screened a panel of TAs preserved in our laboratory for the enzymatic amination of (−)-perillyl aldehyde. ATA-117 from *Arthrobacter* sp. was found to be the most efficient enzyme, exhibiting a high catalytic activity toward (−)-perillyl aldehyde, approximately 2.8 μmol min^−1^ mg^−1^ lyophilized enzyme powders. Therefore, ATA-117 was finally chosen as the transaminase for our work, which was previously reported to be highly productive for the biocatalytic synthesis of sitagliptin after 11 rounds of directed evolution (Gomm et al. 2016; Guan et al. 2015; Savile et al. 2010).

### Optimizing the cascade reactions of Module 1

Subsequently, we tried to improve the reaction efficiency of Module 1. As shown in Table 1, the effects of a few parameters such as temperature, pH, substrate concentration, cell dosage and whether to add surfactants were investigated. The titer of (−)-perillyl alcohol was a little bit higher at pH 7.5, and on this basis, we lowered the reaction temperature to 25 ℃. To our surprise, the concentration of formed (−)-perillyl alcohol was doubled. Strangely, an increase in the cell dosage from 5 to 20 g_cdw_/L resulted in a slight decrease in the analytic yield of the final product. This might be caused by the increase of the viscosity of the reaction system, which may affect the mass transfer rate. To address this problem, we tried to add 1 mg/mL of Triton X-100 to the reaction system. Finally, under the conditions of 15 g_cdw_/L cell dosage, 10 mM substrate and 1 mg/mL Triton X-100, the concentration of product (−)-perillyl alcohol reached 6.8 mM. Partial volatilization of (−)-limonene led to the loss of substrate (Cornelissen et al. 2013), as confirmed in a system without cells, while the remaining (−)-limonene (*ca.* 68%) was completely oxidized into (−)-perillyl alcohol.

**Table 1:** Optimization of reaction conditions of Module 1

Entry	Temp. (°C)	pH	Sub. conc. (mM)	Cell dosage (gcdw/L)	(−)-Perillyl alcohol (mM)
1	30	8.0	5.0	10	1.0
2	30	7.5	5.0	10	1.4
3	30	8.5	5.0	10	0.9
4	25	7.5	5.0	10	2.7
5	25	7.5	5.0	5	2.8
6	25	7.5	5.0	15	2.0
7	25	7.5	5.0	20	1.9
8^a^	25	7.5	5.0	5	1.9
9^a^	25	7.5	5.0	10	2.9
10^a^	25	7.5	5.0	15	4.6
11^a^	25	7.5	5.0	20	2.7
12^a^	25	7.5	7.5	15	5.1
13^a^	25	7.5	10	15	6.8
14^a^	25	7.5	15	15	6.7

Reaction conditions (0.5 mL): ( −)-limonene (5–10 mM), A7F resting cells (5–20 g_cdw_/L), sodium formate (100 mM), DMSO (2% v/v), KPB buffer (pH 7.5, 100 mM) or Tris–HCl buffer (pH 8.0–8.5, 100 mM). The reaction mixtures were shaken at 25 or 30 °C and 800 rpm for 4 h

^a^Additional 1 mg/mL of Triton X-100 was added. Temp.: temperature; Sub. conc.: substrate concentration

### Optimizing the cascade reactions of Module 2

**Fig. 1:**
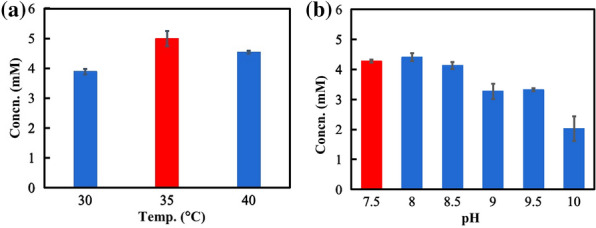
Optimization of the temperature (**a**) and pH (**b**) of Module 2. Reaction conditions (0.5 mL): (−)-Perillyl alcohol 10 mM, *Lk*ADH 0.2 U/mL, [*Lk*ADH]/[NOX]/[ATA-117] = 1/5/10, 0.2 mM NAD^+^, 0.1 mM PLP, 50 mM 2-pentanamine, potassium phosphate buffer (pH 7.5, 100 mM) or Tris–HCl buffer (pH 8–9, 100 mM) or Gly-NaOH buffer (pH 9.5–10, 100 mM), 30℃, 35 ℃ or 40 ℃, 800 rpm, 12 h. Temp.: temperature; Concn.: concentration

In order to optimize the reaction efficiency of Module 2, the effects of a few parameters were examined, including temperature, pH, amine donor and cofactor doses were examined. Based on actual effects on the reaction, the optimal pH and temperature for the reaction were pH 7.5 and 35 ℃ (Fig. 1). In a one-pot reaction, there are multiple enzymes coexisting in the same space. In order to improve the overall efficiency of cascaded reactions, it is important to coordinate the ratio of each element added into the system. We have investigated the ratio of the three enzyme doses in Module 2, suggesting that the optimal dose ratio of [*Lk*ADH]/[*Sm*NOX]/[ATA-117] should be 1:2:10 (Additional file 1: Fig. S1). After determining the proportion of the enzyme added, we examined the effect of enzyme doses. Keeping a constant ratio of the three enzymes, the product titer increased with the increase in the enzyme doses. When the amount of *Lk*ADH was 0.2 U/mL, the product titer was the highest, so the optimum dose of *Lk*ADH was fixed to be 0.2 U/mL (Additional file 1: Fig. S2).

**Fig. 2:**
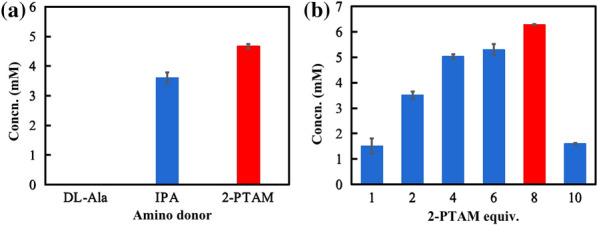
Optimization of the kind (**a**) and equivalents (**b**) of amino donor in Module 2. Reaction conditions (0.5 mL): shaken at 35 °C, 800 rpm for 12 h, KPB buffer (pH 7.5, 100 mM). The reaction mixture (0.5 mL) was composed of 10 mM (−)-perillyl alcohol (with 2% DMSO), 0.2 U/mL *Lk*ADH, 0.4 U/mL *Sm*NOX, 2 U/mL ATA-117, 0.2 mM PLP, 0.2 mM NAD^+^, in addition to: **a** 50 mM DL-Ala/IPA/2-pentanamine; **b** 10−100 mM 2-pentanamine. *IPA* isopropanyl alcohol, *Concn*. Concentration, *2-PTAM* 2-pentanamine, *equiv*. equivalent

Transaminases have the significant advantages that do not require redox cofactors, however the unfavorable thermodynamic equilibrium of enzymes limits its application (Kelefiotis-Stratidakis et al. 2019). The amino donor may be an important factor to overcome the shortcomings of reaction equilibrium. We optimized the type and amounts of amino donors. Among several commonly used amine donors, 2-pentanamine is the most effective one (Fig. 2A). In previous studies, researchers usually added excessive amounts of amino donor to shift the equilibrium [14], but excessive amino donor will also impair the activity of enzyme. The product titer was the highest when 80 mM 2-pentanamine was added to the reaction system (Fig. 2B). In addition, the doses of cofactors NAD^+^ and pyridoxal-5-phosphate (PLP) were also optimized, indicating that 0.2 mM NAD^+^ and 0.2 mM PLP were sufficient for the cascaded reaction (Additional file 1: Fig. S3). Through the optimization of Module 2, the titer of (−)-perillamine was increased up to nearly 7 mM.

### One-pot two-step cascade reactions

Initially, we tried one-pot one-step and one-pot two-step modes to perform this cascade reaction, respectively. When we performed the cascaded reaction in the one-pot one-step mode, only a trace amount of the final product (−)-perillamine was detected. The efficiency of the cascade reaction was relatively low, implying that the one-pot one-step mode might be not conducive to the catalytic reaction of unstable P450 enzyme in the complicated reaction system. Therefore, the cascade reactions were alternatively performed in a one-pot two-step mode. When the cascaded reactions of Module 1 were complete, the reaction mixture was heated in a 65 °C water bath to inactivate formate dehydrogenase to avoid any interference with the subsequent coenzyme recycling. All the elements desired for Module 2 were supplemented into the reaction system after being cooled to room temperature and the incubation was continued for 12 h. In the one-pot two-step mode, the titer of (−)-perillamine had a slight increase to approximately 2 mM, suggesting that there was another bottleneck existing in our cascade reaction system. Through step-by-step investigation, we found that only a trace amount of (−)-perillyl aldehyde could be detected in the dehydrogenation step catalyzed by *Lk*ADH. By measuring the activity of *Sm*NOX, it turned out that the surfactant added in Module 1 had negative effects on the enzyme activity of *Sm*NOX (Additional file 1: Table S1) and the recycling efficiency of the cofactor. Therefore, we had to increase the *Sm*NOX dose from 0.2 to 1 U/mL for a compensation, which resulted in a significant increase of the product titer.

### 100-mL preparative-scale cascade reaction

**Fig. 3:**
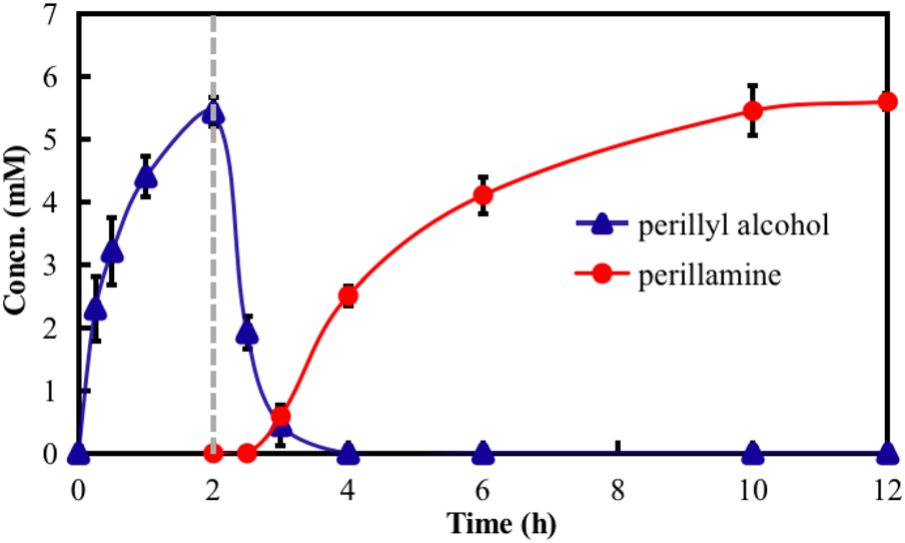
Time course of the 100-mL preparative-scale multi-enzyme cascade reaction. Step I: 10 mM (−)-limonene (with 2% DMSO), 15 g_cdw_ L^−1^ A7F resting cells, 0.2 mM NAD^+^, 100 mM sodium formate, 1 mg/mL Triton X-100, KPB buffer (100 mM, pH 7.5), 25 °C, 200 rpm. Step II: supplement of 0.2 U mL^−1^ *Lk*ADH, 1 U mL^−1^ *Sm*NOX, 2 U mL^−1^ ATA-117, 80 mM 2-pentanamine, 0.2 mM NAD^+^ and 0.2 mM PLP, incubated at 35 °C and shaken at 200 rpm. Concn.: concentration

Under the optimized conditions, we carried out a 100 mL preparative-scale cascade reaction, where the time-course analysis revealed that the formation of (−)-perillamine reached a maximum 5.4 mM at approximately 12 h (Fig. 3). Since monoterpenoids are extremely volatile, it was difficult to accurately determine their content. We only monitor the concentrations of the intermediate product (−)-perillyl alcohol, and the final product (−)-perillamine, which are relatively not easy to be volatilized. The reaction of Module 1 quickly reached the end point in 2 h, and there was no significant increase in product titer when the reaction time was prolonged. After the reaction mixture of Module 2 was supplemented, the resulting (−)-perillyl alcohol in Module 1 was rapidly converted into (−)-perillyl aldehyde, accompanied by the formation of (−)-perillamine as the final product. The product was isolated from the reaction system and purified by silica gel column chromatography, giving 76 mg (−)-perillamine with a space–time yield of 1.5 g L^−1^ d^−1^. ^1^H-NMR (400 MHz, CDCl_3_) δ/ppm (Additional file 1: Fig. S4): 5.58 (brs, 1H), 4.71 (brs, 2H), 3.17 (brs, 2H), 2.19–1.85 (m, 7H), 1.73 (s, 3H), 1.53–1.40 (m, 1H). ^13^C NMR (101 MHz, CDCl_3_) δ/ppm: 149.97, 138.50, 120.22, 108.56, 47.92, 41.28, 30.50, 27.64, 27.12, 20.81.

## Conclusions

In summary, by recruiting hydroxylase, alcohol dehydrogenase, transaminase, formate dehydrogenase and NADH oxidase, a redox self-sufficient and concise multi-enzyme cascade reaction was successfully constructed to transform the cheap and easily available (−)-limonene into (−)-perillamine for the first time. After optimization of the two modules, 10 mM loaded (−)-limonene was smoothly converted into 5.4 mM (−)-perillamine in 12 h. Our work is of great significance for the development of new biologically active compounds. More works of molecular evolution, protein engineering and process optimization may be necessary to further improve the performance of the one-pot biotransformation and to minimize the waste of the excessive co-substrate. It is envisioned that this elegant enzymatic route might be applied for bioamination of many other terpenoids, such as pinene, geraniol, citronellol, etc., especially for the synthesis of the biologically active compounds with promising application prospects.

### Supplementary Information

**Additional file 1: Figure S1.** Optimization of the ratio of each elemental enzyme added in Module 2. **Figure S2.** Optimization of enzyme dose. **Figure S3.** Optimization of cofactor dose. **Figure S4.** ^1^H-NMR spectrum of pure (−)-perillamine. **Table S1.** Inactivation effect of Triton X-100 on *Sm*NOX activity.

## Data Availability

All data generated or analyzed during this study are included in this article and its Additional files.

## Abbreviations

*Cb*FDH: Formate dehydrogenase from *Candida boidinii*
*Lk*ADH: Alcohol dehydrogenase from *Lactobacillus kefiri*
*Sm*NOX: NADH oxidase from *Streptococcus mutans*
ATA-117: ω-Transaminase from *Arthrobacter* sp.
NADH: Nicotinamide adenine dinucleotide
PLP: Pyridoxal-5-phosphate
