# Supplementary material for: Regiospecific C–H amination of (−)-limonene into (−)-perillamine by multi-enzymatic cascade reactions
Source: Bioresour Bioprocess. 2022 Aug 26;9(1):88. doi: 10.1186/s40643-022-00571-x (PMC10992285; doi:10.1186/s40643-022-00571-x)
Supplement: Supplementary file 1 — Additional file 1: Figure S1. Optimization of the ratio of each elemental enzyme added in Module 2. Figure S2. Optimization of enzyme dose. Figure S3. Optimization of cofactor dose. Figure S4. 1H-NMR spectrum of pure (−)-perillamine. Table S1. Inactivation effect of Triton X-100 on SmNOX activity. [file 40643_2022_571_MOESM1_ESM.docx]

Supporting Information for

**Regiospecific C-H Amination of (─)-Limonene into (─)-Perillamine by Multi-enzymatic Cascade Reactions**

**Yue Ge, Zheng-Yu Huang, Jiang Pan, Chun-Xiu Li, Gao-Wei Zheng and Jian-He Xu***

Laboratory of Biocatalysis and Synthetic Biotechnology, State Key Laboratory of Bioreactor Engineering, Shanghai Collaborative Innovation Centre for Biomanufacturing, College of Biotechnology, East China University of Science and Technology, Shanghai 200237, P. R. China.

* Corresponding authors: Prof. Jian-He Xu. E-mail: jianhexu@ecust.edu.cn

**Figure S1.** Optimization of the ratio of each elemental enzyme added in Module 2. Reaction conditions (0.5 mL system): KPB buffer (100 mM, pH 7.5), shaken at 35 ℃, 800 rpm for 12 h, 10 mM (−)-perillyl alcohol (with 2% DMSO), 0.2 mM PLP, 0.2 mM NAD^+^, and 80 mM 2-pentanamine, in addition to: a) 0.2 U/mL *Lk*ADH, 2 U/mL ATA-117 and varied doses of *Sm*NOX; b) 0.2 U/mL *Lk*ADH, 0.4 U/mL *Sm*NOX and varied doses of ATA-117. Concn.: concentration.

**Figure S2.** Optimization of enzyme dose of *Lk*ADH. Reaction conditions: (-)-perillyl alcohol, 10 mM; 0.1, 0.2 or 0.3 U/mL *Lk*ADH; [*Lk*ADH]/[NOX]/[ATA-117] = 1/5/10; 0.2 mM NAD^+^; 0.2 mM PLP; 80 mM 2-pentanamine; KPB buffer (100 mM, pH 7.5); 35℃, 800 rpm; 12 h.

**Figure S3.** Optimization of cofactor dose. Reaction conditions: (-)-perillyl alcohol, 10 mM; *Lk*ADH, 0.2 U/mL; [*Lk*ADH]/[NOX]/[ATA-117] = 1/5/10; NAD^+^, 0, 0.2, 0.4, or 0.6 mM; PLP, 0.1, 0.2, 0.4, or 0.6 mM; 2-pentanamine, 80 mM; KPB buffer, 100 mM (pH 7.5); Incubation, 35 ℃, 800 rpm, 12 h.


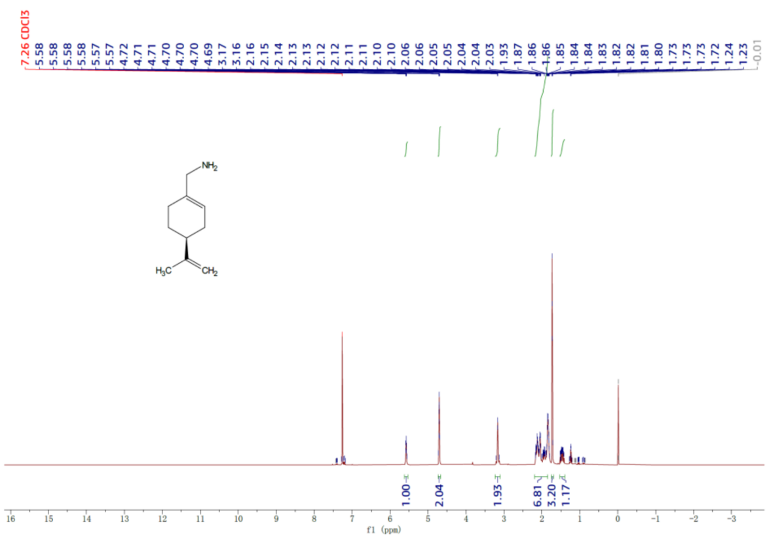


**Figure S4.** ^1^H-NMR spectrum of the purified (-)-perillamine from the multi-enzymatic reaction mixture.

**Table S1.** Inactivation effect of Triton X-100 on the *Sm*NOX activity.

| **Enzyme** | **Residual activity (%)** | |
| --- | --- | --- |
|  | 0 h | 12 h |
| *Sm*NOX | 100 | 81 ± 4 |
| *Sm*NOX + Triton X-100 | 94 ± 1 | 37 ± 3 |

The experiments were performed for triplicates and shown as the average ± standard deviations (SD).
